# Development of a midlife-specific CogDrisk algorithm (CogDrisk-ML) to enable validated implementation of dementia risk assessment from midlife to late life

**DOI:** 10.1093/ageing/afaf201

**Published:** 2025-07-21

**Authors:** Md Hamidul Huque, Heidi Jane Welberry, Ranmalee Eramudugolla, Nicola T Lautenschlager, Kaarin J Anstey

**Affiliations:** School of Psychology, University of New South Wales, Sydney, NSW, Australia; Brain Health and Dementia Centre, Neuroscience Research Australia, Sydney, NSW, Australia; UNSW Ageing future Institute, University of New South Wales, Sydney, Australia; Centre for Big Data Research in Health, University of New South Wales, Sydney, NSW, Australia; School of Psychology, University of New South Wales, Sydney, NSW, Australia; Brain Health and Dementia Centre, Neuroscience Research Australia, Sydney, NSW, Australia; UNSW Ageing future Institute, University of New South Wales, Sydney, Australia; Academic Unit for Psychiatry of Old Age, Department of Psychiatry, University of Melbourne, Melbourne, VIC, Australia; Older Adult Mental Health Program, Royal Melbourne Hospital Mental Health Service, Melbourne, VIC, Australia; School of Psychology, University of New South Wales, Sydney, NSW, Australia; Brain Health and Dementia Centre, Neuroscience Research Australia, Sydney, NSW, Australia; UNSW Ageing future Institute, University of New South Wales, Sydney, Australia

**Keywords:** risk tool, risk assessment, dementia prediction, dementia prevention, midlife risk factors, older people

## Abstract

**Background:**

Existing dementia risk assessment tools, such as The Australian National University Alzheimer’s Disease Risk Index (ANU-ADRI), LIfestyle for BRAin health (LIBRA) and Cognitive health and Dementia Risk Assessment (CogDrisk), show limited validation for middle-aged adults (age 40–64 years). The Cardiovascular Risk Factors, Aging, and Incidence of Dementia (CAIDE) tool, developed almost two decades ago, demonstrated moderate predictive accuracy. As key modifiable dementia risk factors emerge in midlife, there is a need for a new, more accurate midlife dementia risk assessment tool.

**Objectives:**

To develop CogDrisk-ML, a midlife dementia risk assessment tool that can complement the existing CogDrisk tool for late-life dementia risk assessment.

**Design and Settings:**

Data from the UK Biobank and the Atherosclerosis Risk in Communities (ARIC) study were used to develop and validate CogDrisk-ML, which was also externally validated using the Whitehall II cohort.

**Participants and Covariates:**

Participants without dementia at baseline were included, with CogDrisk predictors along with additional midlife risk factors based on recent evidence.

**Main Outcome Measures:**

Cox regression models estimated the relationship between risk factors and dementia for each sex. A random-effects meta-analysis model aggregated cohort- and sex-specific regression coefficients to develop CogDrisk-ML. Harrell’s *C* statistics measured predictability, with multiple imputation used for missing data.

**Results:**

CogDrisk-ML outperformed CAIDE in the UK Biobank and Whitehall II cohorts; however, it provided similar *C* statistics in the ARIC dataset. *C* statistics (95% confidence interval) for CogDrisk-ML were 0.71 (0.69, 0.74) for the ARIC, 0.75 (0.73, 0.77) for the UK Biobank and 0.70 (0.62, 0.79) for the Whitehall II study.

**Conclusion:**

The novel CogDrisk-ML for assessing dementia risk in midlife offers improved predictive accuracy. Combined with the CogDrisk tool for late life, it provides a comprehensive framework for dementia prevention throughout the life course.

## Key Points

- We developed CogDrisk-ML, which can be implemented alongside currently available late-life dementia risk assessment tools.
- This tool expands CogDrisk to enable implementation throughout the entire life course from midlife to late life.
- It provides a comprehensive framework for dementia prevention that can be used by general practitioners and policy-makers.

## Introduction

Dementia, an urgent global health priority, is a leading cause of disability and dependency among older adults [1]. To date, there is no effective and widely available treatment for dementia. As a result, dementia risk scores are currently being used as a prevention strategy to improve risk perception for patients and clinicians. These scores help early identification of individuals with modifiable risk factors, enabling healthcare professionals to recommend targeted interventions that improve lifestyle, manage medical conditions and decrease dementia risk.

Recent evidence suggests that most of the key modifiable risk factors of dementia emerge in midlife, and neuropathological changes that lead to dementia often emerge in midlife as well [2, 3]. The Lancet Commission Report (2024) identified 14 modifiable risk factors, which, with appropriate interventions, could prevent up to 45% of dementia cases [4, 5]. These include early life education, and midlife risk protective factors such as obesity, excessive alcohol consumption, hearing loss, high LDL cholesterol, depression, traumatic brain injury (TBI), physical inactivity, smoking, diabetes and hypertension [4]. Evidence from systematic reviews and empirical studies [4, 6, 7] suggests that proper management of comorbidities, e.g. hypertension, stroke, diabetes, TBI and depression, and modification of lifestyle factors, such as physical activity, sleep, diet and smoking, can improve cognitive impairment.

Several risk scores, including ANU-ADRI [8], LIBRA [9] and CogDrisk [10], have been developed using meta-analysis of quantitative data from existing literature. However, validation of these risk scores for estimating risk of dementia in middle-aged adults (aged between 40 and 64 years) is limited. When applied to individual risk assessment in midlife (age between 40 and 64, inclusive), these risk scores show low validation accuracy [area under the curve (AUC): <0.60] [11–13]. CAIDE [14] is the only risk assessment tool that was developed using midlife population from a single cohort; however, it demonstrated only moderate predictive accuracy when validated in independent cohort data (AUC: 0.60–0.69) [12, 13]. Moreover, CAIDE was developed nearly two decades ago and therefore does not include the most current knowledge of risk factors. Therefore, development of a new midlife dementia risk score is critically important for dementia prevention.

The lack of risk scores for middle-aged adults has been primarily due to the lack of available datasets that include middle-aged adults who have been followed long enough to obtain incident dementia diagnoses. However, the recent release of new datasets, including the UK Biobank [13], the Whitehall II study [14] and the Biologic Specimen and Data Repository Information Coordinating Center (BioLINCC) within National Institute of Health (NIH), offer excellent datasets that assessed midlife information on the risk factors and later-life dementia outcomes. A risk assessment tool that generates dementia risk scores using midlife risk or protective factors from multiple countries will be more generalizable and acceptable to general practitioners (GPs), the public and policy-makers than one built using data from a single country.

To date, the available risk assessment tools address dementia risk discretely at different life stages (i.e. midlife or late life). Therefore, a single tool validated for use in both midlife and late life and thus covering the full life course of an individual would be a more practical approach for clinicians and individuals. In this paper, we therefore developed a midlife risk assessment tool using longitudinal data from multiple countries that can be used in combination with other existing risk assessment tools for late life.

Of the existing dementia risk assessment tools, the CogDrisk risk score [10] covers the widest range of established contemporary factors for increased or reduced risk of dementia, including education, obesity, diabetes, high cholesterol, hypertension, stroke, TBI, insomnia, depression, physical inactivity, cognitive activities, social engagement, diet and smoking. It has also been externally validated using high-quality international cohort studies to predict dementia [11, 15] and Alzheimer's disease (AD) [16], implemented online using an interactive website [17], incorporated into research studies [18, 19] and gained popularity among GPs and the general public [20]. In addition to the risk and protective factors available in the CogDrisk tool, the Lancet Commission’s 2024 [4] report identified ‘hearing loss’ as an important midlife risk factor for dementia. Therefore, we developed CogDrisk-ML by incorporating hearing loss, along with all the available midlife risk factors of CogDrisk, hence labelled ‘CogDrisk-ML’.

## Methods

In this cohort study, we considered data from the UK Biobank and the Atherosclerosis Risk in Communities (ARIC) population-based cohorts with dementia for the development/validation of the CogDrisk-ML risk tool for dementia. In addition, we also considered the Whitehall II study for external validation. The study was conducted between March 2024 and November 2024 and followed the Strengthening the Reporting of Observational Studies in Epidemiology (STROBE) reporting guideline.

### Population-based cohorts for development and validation of CogDrisk-ML

#### The UK Biobank

The UK Biobank is a longitudinal cohort study of over 500 000 individuals aged 40–69 years, recruited between 2006 and 2010 across the UK [21]. Participants were followed up via linked electronic health records from the UK National Health Service (NHS), with up to 15 years of follow-up data available for our analysis, until 2022. Dementia diagnoses were ascertained using multiple linked datasets, including self-reported medical history, primary care records, hospital inpatient records and death registry records [22]. For this analysis, we excluded 608 individuals who had a dementia diagnosis before the age of 65 years (i.e. early-onset dementia). We therefore restricted our samples to individuals aged 50–64 years at baseline (*n* = 288 552). This research was conducted under UK Biobank application number 79620.

#### Atherosclerosis Risk in Communities

The ARIC cohort study was conducted in four geographically diverse US communities. The study included 15 792 participants aged 45–65 years at their baseline (1987–89) [23]. While the study is still ongoing, the dementia was first assessed during exam 5 (2011–13). For this analysis, we restrict our analysis to participants who had dementia assessment during wave 5 and who were aged < 65 years at baseline (*n* = 5860). This dataset does not contain any cases of early onset of dementia. Dementia was diagnosed using an algorithm and ICD-9 codes, following the Diagnostic and Statistical Manual of Mental Disorders (DSM-III-R) criteria [24]. All the participants included in our analysis from the ARIC dataset had a follow-up period of ~29 years (from exam 1 to exam 7). The dataset was obtained using BioLINCC (application number 13630).

#### Whitehall II

Whitehall II is a longitudinal cohort of 10 308 British civil servants aged 35–55 at baseline, recruited between 1985 and 1988. A total of 12 waves of data collections are available, with wave 12 conducted during 2015–16. To ensure consistency with the UK Biobank and ARIC cohorts in terms of age composition, we selected wave 5 (conducted in 1997–99) as the baseline for this analysis. Wave 5 also includes most of the variables necessary for calculating CogDrisk-ML. In total, 6516 participants of age 45–65 were included in the analysis. Dementia was defined based on self-reported diagnosis. The dataset was obtained and analysed through Dementia Platform UK (application number 704).

Detailed definitions of dementia outcomes and risk/protective factors and their availability are provided in Appendix S1 in the Supplementary Data section. It is important to note that none of the development datasets used in this analysis contain cognitive activity, and therefore cognitive activity was not included in the development of CogDrisk-ML.

### Statistical analysis

To develop CogDrisk-ML, the UK Biobank and ARIC datasets were randomly split into two parts in 60:40 ratios, with 60% used for model development (model data) and 40% for validation. In addition, we used the full Whitehall II dataset for external validation. Proportionate representation of age, sex and education was ensured in the development and validation samples. Descriptive statistics were calculated.

Both model data cohorts contained missing values for several risk and protective factors. To address missing data in the model dataset, we performed multiple imputation analysis using multivariate normal imputation (MVNI) [25]. We considered congenial imputation models that included all the relevant covariates and outcomes of interest [26]. Ten imputed datasets were generated following von Hippel *et al.*’s guidelines [27]. For each sex, Cox proportional hazards regression models were fitted to each imputed dataset to estimate regression coefficients. The estimated regression coefficients from these datasets were then combined using Rubin’s rule to obtain sex-specific regression coefficients for each risk factor. These sex- and cohort-specific regression coefficients for each of the risk factors were further aggregated across the UK Biobank and ARIC cohorts using random-effects meta-analysis. To implement CogDrisk-ML with the CogDrisk tool, the model includes regression coefficients of all the predictors, irrespective of their statistical significance. The final regression coefficients were then converted to obtain point-based CogDrisk-ML scoring algorithms for each sex.

The resultant CogDrisk-ML scores were then validated using validation data using both complete case analysis and analysis following multiple imputation. Model calibration was conducted using the Hosmer–Lemeshow goodness-of-fit test along with a calibration plot using validation data [28]. The accuracy of the risk scores for identifying participants at risk of dementia was also determined by calculating Harrell’s *C* statistics [29] and the associated 95% confidence intervals (CIs). Cutoff values (quantile ranks) for the risk scores were compared relative to hazards ratios, and for sensitivity and specificity. Similar to the development dataset, MVNI was applied to handle missing data in the validation datasets.

In addition to the validation of CogDrisk-ML, we conducted two analyses to investigate the role of age in the performance of dementia risk scores. First, we compared the performance of CogDrisk-ML with CAIDE and the original CogDrisk when the risk scores were calculated without including age weights. Second, we compared the performance of CogDrisk-ML with an age- and sex-only model. For the comparison of CAIDE, and CogDrisk, with CogDrisk-ML, the same procedure was applied: the points associated with each available risk factor in the respective cohort were summed to calculate each risk score [15]. The performance of these risk scores was also determined using Harrell’s *C* statistics. All the analyses were conducted using Stata statistical software version 16.0 (Stata Corp).

## Results

### Description of the study cohort

Appendix S2 shows the distribution of covariates and outcomes in the model development and validation datasets. There are some key differences across the study datasets. Although similar sex distributions were observed for the UK Biobank and ARIC datasets (male 42%–44%), the Whitehall study had a predominantly male sample (71%). Participants in the Whitehall study were highly educated, with the highest proportion of tertiary educated participants (81%), followed by the UK Biobank (62%) and ARIC (44%). Prevalence of hypertension and high cholesterol was similar across all the cohorts, while obesity and diabetes were more common in the ARIC and UK Biobank studies than in the Whitehall study. The proportion of current smokers was similar in UK Biobank and Whitehall, whereas ARIC had a higher proportion. The ARIC participants were followed for almost 30 years, whereas UK Biobank and Whitehall had follow-up periods of 13 and 17 years, respectively. Approximately 20% of the participants in our analysis of the ARIC dataset were diagnosed with dementia, compared to 0.7% and 0.8% in the UK Biobank and Whitehall datasets, respectively.

### Development and validation of CogDrisk-ML

Table 1 shows the estimated regression coefficients and 95% CIs from the Cox regression model using model development data, along with corresponding points for the CogDrisk-ML score. For males, higher age, lower education, loneliness and having had hypertension, depression, diabetes, stroke and sleep problems are significantly associated with dementia risk. For females, significant dementia risk is associated with less than tertiary education, smoking, loneliness and having had high cholesterol, depression, diabetes and hearing loss. See Appendix S3 in the Supplementary Data section for the full details of cohort-specific estimates. In both cohorts, higher age, lower education and diabetes are significantly associated with increasing risk of dementia for males. For females, only age and diabetes are significant.

**Table 1:** Pooled regression coefficients and associated point estimates of each of the risk factors for the midlife CogDrisk score.

	Pooled regression coefficients $\beta\ \left(95\%\mathrm{CI}\right)$		Point estimates	
Covariates	Male	Female	Male	Female
Age group				
44–49	−0.33 (−0.69, 0.02)	−0.81 (−1.11, −0.51)	−3	−8
50–54	Ref.	Ref.	0	0
55–59	1.61 (−0.10, 3.32)	2.29 (−1.56, 6.15)	16	23
60–64	2.63 (0.16, 5.09)	3.34 (−1.00, 7.68)	26	33
Education				
0 to <8 years	0.58 (0.28, 0.88)	0.36 (0.02, 0.71)	6	4
8–11 years	0.16 (−0.27, 0.59)	0.20 (0.05, 0.35)	2	2
>11 years	Ref.	Ref.	0	0
Obesity				
Underweight	0.43 (−0.70, 1.55)	0.48 (−0.16, 1.12)	4	5
Normal weight	Ref.	Ref.	0	0
Overweight	−0.13 (−0.50, 0.24)	0.16 (−0.13, 0.44)	−1	2
Obese	−0.29 (−0.54, −0.05)	−0.03 (−0.28, 0.23)	−3	0
Smoking status				
Never smoker	Ref.	Ref.	0	0
Former smoker	−0.04 (−0.19, 0.11)	0.07 (−0.08, 0.22)	0	1
Current smoker	0.15 (−0.07, 0.37)	0.38 (0.17, 0.59)	2	4
Hypertension (yes vs no)	0.19 (0.03, 0.34)	0.14 (−0.02, 0.29)	2	1
High cholesterol (yes vs no)	0.14 (−0.32, 0.61)	0.18 (0.03, 0.33)	1	2
Depression (yes vs no)	0.47 (0.14, 0.80)	0.42 (0.13, 0.70)	5	4
Fish serve	−0.28 (−0.56, 0.00)	0.08 (−0.10, 0.26)	−3	1
Physical activity (less than sufficient vs sufficient)	0.15 (−0.01, 0.32)	0.05 (−0.11, 0.22)	2	1
Diabetes (yes vs no)	0.86 (0.65, 1.07)	0.68 (0.42, 0.95)	9	7
Stroke (yes vs no)	1.10 (0.75, 1.45)	0.58 (−0.86, 2.03)	11	6
Traumatic brain injury (yes vs no)	0.39 (−0.52, 1.30)	0.28 (−0.41, 0.97)	4	3
Loneliness (yes vs no)	0.33 (0.10, 0.56)	0.32 (0.10, 0.55)	3	3
Sleep problem (Yes vs No)	0.19 (0.00, 0.38)	−0.26 (−0.46, −0.06)	2	−3
Hearing loss (yes vs no)	0.08 (−0.07, 0.22)	0.24 (0.09, 0.39)	1	2

Table 2 shows Harrell’s *C* statistics with 95% CIs for estimating dementia risk with CogDrisk-ML and CAIDE across all three validation datasets, obtained using both complete case analysis and analysis followed by multiple imputation. In the complete case analysis, the CogDrisk-ML shows reasonable estimates, with the overall *C* statistics (95% CIs) being 0.71 (0.69, 0.74) for ARIC, 0.75 (0.73, 0.77) for UK Biobank and 0.70 (0.62, 0.79) for Whitehall II. Similar estimates of *C* statistics were obtained for both males and females. CogDrisk-ML provides similar estimated *C* statistics to the CAIDE risk score in the ARIC dataset [CogDrisk-ML: 0.71 (0.69, 0.74); CAIDE 0.69 (0.67, 0.72)]. However, CogDrisk-ML shows better performance when applied to the UK Biobank [CogDrisk-ML: 0.75 (0.73, 0.77); CAIDE: 0.60 (0.57, 0.62)] and Whitehall II [CogDrisk-ML: 0.70 (0.62, 0.79); CAIDE: 0.53 (0.43, 0.62)] datasets. The calibration plots (see Appendix S4 in the Supplementary Data section) show that CogDrisk-ML provides good calibration for predicting dementia for both males and females in validation datasets. Multiple imputation of the missing data yielded similar *C* statistics compared to complete case analysis (Table 2).

**Table 2:** Estimated Harrell’s *C* statistics (95% CI) of CogDrisk-ML and CAIDE risk scores across three datasets.

		Complete case analysis		
		ARIC data *M* = 933, *F* = 1263, *T* = 2196	UK-Biobank *M* = 33 174, *F* = 37 349, *T* = 70 523	Whitehall II *M* = 2923, *F* = 1106, *T* = 4029
CogDrisk-ML	Male	0.71 (0.68, 0.75)	0.76 (0.74, 0.79)	0.71 (0.60, 0.81)
	Female	0.73 (0.70, 0.76)	0.77 (0.74, 0.79)	0.68 (0.51, 0.85)
	Overall	0.71 (0.69, 0.74)	0.75 (0.73, 0.77)	0.70 (0.62, 0.79)
				
CAIDE	Male	0.71 (0.67, 0.74)	0.59 (0.56, 0.62)	0.50 (0.38, 0.60)
	Female	0.68 (0.64, 0.71)	0.58 (0.54, 0.62)	0.55 (0.40, 0.70)
	Overall	0.69 (0.67, 0.72)	0.60 (0.57, 0.62)	0.53 (0.43, 0.62)
		Multiple imputed datasets		
		*M* = 985, *F* = 1353, *T* = 2338	*M* = 42 248, *F* = 52,425, *T* = 94 673	*M* = 4614, *F* = 1902, *T* = 6516
CogDrisk-ML	Male	0.72 (0.68, 0.76)	0.76 (0.74, 0.78)	0.69 (0.61, 0.77)
	Female	0.74 (0.71, 0.78)	0.77 (0.75, 0.79)	0.67 (0.55, 0.79)
	Overall	0.73 (0.70, 0.75)	0.72 (0.71, 0.74)	0.68 (0.62, 0.74)
				
CAIDE	Male	0.71 (0.67, 0.75)	0.58 (0.55, 0.61)	0.53 (0.45, 0.60)
	Female	0.70 (0.67, 0.74)	0.58 (0.55, 0.61)	0.59 (0.49, 0.69)
	Overall	0.71 (0.68, 0.73)	0.59 (0.57, 0.61)	0.55 (0.49, 0.62)

M, F, and T represents sample size for Male, Female, and Total, respectively.

### The role of age in risk scores for predicting dementia

Table 3 compares the performance of CogDrisk-ML, CAIDE and CogDrisk following complete case and multiple imputation analysis, where the risk scores were calculated without including age and sex weights. The overall performance of all three risk scores is similar across the datasets, with higher *C* statistics (95% CI) observed in the ARIC dataset, followed by the UK Biobank and Whitehall II datasets. All the risk scores performed poorly in the Whitehall dataset for females. Imputed data resulted in similar estimates to the complete case analysis.

**Table 3:** Estimated Harrell’s *C* statistics (95% CI) of CogDrisk-ML, CAIDE and CogDrisk scores across three datasets, where these scores are calculated without including age effect in the model.

	Complete case analysis			
		ARIC data *M* = 933, *F* = 1263, *T* = 2196	UK-Biobank *M* = 33 174, *F* = 37,349, *T* = 70 523	Whitehall II *M* = 2923, *F* = 1106, *T* = 4029
CogDrisk-ML	Male	0.63 (0.59, 0.68)	0.60 (0.56, 0.63)	0.62 (0.50, 0.73)
	Female	0.66 (0.62, 0.70)	0.57 (0.53, 0.61)	0.52 (0.34, 0.70)
	Overall	0.61 (0.58, 0.64)	0.59 (0.56, 0.62)	0.53 (0.42, 0.64)
				
CAIDE	Male	0.66 (0.61, 0.70)	0.56 (0.53, 0.59)	0.54 (0.43, 0.65)
	Female	0.62 (0.58, 0.66)	0.55 (0.51, 0.59)	0.52 (0.36, 0.68)
	Overall	0.64 (0.61, 0.66)	0.55 (0.53, 0.58)	0.48 (0.38, 0.57)
				
CogDrisk	Male	0.65 (0.61, 0.69)	0.54 (0.51, 0.58)	0.52 (0.40, 0.64)
	Female	0.64 (0.60, 0.67)	0.54 (0.50, 0.58)	0.51 (0.37, 0.66)
	Overall	0.64 (0.61, 0.66)	0.54 (0.52, 0.57)	0.51 (0.41, 0.60)
		Multiple imputed datasets		
		*M* = 985, *F* = 1353, *T* = 2338	*M* = 42 248, *F* = 52 425, *T* = 94 673	*M* = 4614, *F* = 1902, *T* = 6516
CogDrisk-ML	Male	0.63 (0.59, 0.67)	0.59 (0.56, 0.62)	0.62 (0.54, 0.71)
	Female	0.66 (0.62, 0.70)	0.58 (0.54, 0.61)	0.52 (0.40, 0.64)
	Overall	0.62 (0.58, 0.65)	0.57 (0.54, 0.59)	0.57 (0.49, 0.65)
				
CAIDE	Male	0.66 (0.62, 0.70)	0.55 (0.52, 0.58)	0.50 (0.42, 0.58)
	Female	0.63 (0.59, 0.66)	0.55 (0.52, 0.58)	0.56 (0.46, 0.67)
	Overall	0.64 (0.61, 0.67)	0.56 (0.54, 0.58)	0.52 (0.45, 0.58)
				
CogDrisk	Male	0.63 (0.59, 0.67)	0.55 (0.52, 0.58)	0.59 (0.51, 0.67)
	Female	0.65 (0.61, 0.69)	0.55 (0.52, 0.58)	0.55 (0.44, 0.66)
	Overall	0.64 (0.61, 0.67)	0.55 (0.53, 0.57)	0.57 (0.50, 0.64)

M, F, and T represents sample size for Male, Female, and Total, respectively.

Table 4 presents a comparison of CogDrisk-ML with the age- and sex-only model. When compared to the age- and sex-only model, for the midlife datasets with relatively short follow-up periods (i.e. the UK Biobank and Whitehall II studies), the age- and sex-only model yielded *C* statistics comparable to those of CogDrisk-ML. However, for the ARIC dataset, the age-only model provided a distinctively lower *C* statistic [0.66 (0.63, 0.69)].

**Table 4:** Estimated Harrell’s *C* statistics (95% CI) of CogDrisk-ML with age- and sex-only model.

		ARIC data *M* = 933, *F* = 1263, *T* = 2196	UK Biobank *M* = 33 174, *F* = 37,349, *T* = 70 523	Whitehall II *M* = 2923, *F* = 1106, *T* = 4029
CogDrisk-ML	Male	0.71 (0.68, 0.75)	0.76 (0.74, 0.79)	0.71 (0.60, 0.81)
	Female	0.73 (0.70, 0.76)	0.77 (0.74, 0.79)	0.68 (0.51, 0.85)
	Overall	0.71 (0.69, 0.74)	0.75 (0.73, 0.77)	0.70 (0.62, 0.79)
				
Age- and sex-only model	Male	0.66 (0.62, 0.70)	0.73 (0.71, 0.75)	0.74 (0.63, 0.84)
	Female	0.66 (0.63, 0.70)	0.75 (0.73, 0.77)	0.69 (0.56, 0.82)
	Overall	0.66 (0.63, 0.69)	0.72 (0.71, 0.73)	0.73 (0.65, 0.81)

M, F, and T represents sample size for Male, Female, and Total, respectively.

### Sensitivity, specificity, and cutoff points of CogDrisk-ML

Table 5 presents hazard ratios (HRs), sensitivity and specificity with 95% CIs with respect to quantile cutoff values for CogDrisk-ML score across the three cohorts. Overall, compared to the first quantile cutoff, HRs for CogDrisk-ML gradually increased with higher quantile cutoffs across all the datasets. Although the exact risk scores for quantile cutoffs varied between cohorts, a median cutoff (≥50%) provided ~70% sensitivity for ARIC data and 80% for the UK Biobank and Whitehall cohorts. Additionally, a cutoff ≥ 66.6% provided ~60% sensitivity and 70% specificity for all three cohorts.

**Table 5:** Sensitivity and specificity and cutoff points of CogDrisk-ML in the development and validation data.

			Development data			Validation data		
Dataset (score range)	Cutoff (%)	Cutoff score	HR (95% CI)^a^	Sensitivity, % (95% CI)	Specificity, % (95% CI)	HR (95% CI)^a^	Sensitivity, % (95% CI)	Specificity, % (95% CI)
ARIC data Model: −9, 52 Validation: −9, 51	≥16.6	−2	1.09 (0.73, 1.65)	92.7 (90.5, 94.5)	23.0 (21.4, 24.6)	1.97 (1.15, 3.38)	94.0 (91.2, 96.1)	25.1 (23.1, 27.1)
	≥33.3	1	1.91 (1.35, 2.72)	86.8 (81.4, 89.2)	39.6 (37.8, 41.5)	3.67 (2.28, 5.90)	86.8 (83.0, 89.9)	40.9 (38.6, 43.2)
	≥50	5	3.58 (2.59, 4.96)	75.3 (71.9, 78.5)	58.7 (56.8, 60.5)	4.44 (2.76, 7.14)	72.3 (67.6, 76.6)	58.4 (56.1, 60.7)
	≥66.6	13	4.96 (3.63, 6.78)	57.1 (53.3, 60.8)	73.8 (72.1, 75.4)	6.39 (4.09, 9.98)	57.5 (52.5, 62.4)	72.9 (70.8, 75.0)
	≥83.3	28	8.29 (6.12, 11.24)	32.4 (29.0, 36.0)	89.2 (88.0, 90.3)	11.70 (7.57, 18.10)	32.8 (28.2, 37.6)	88.9 (87.3, 90.3)
								
UK Biobank model: −6, 63 Validation: −6, 57	≥16.6	3	7.50 (2.24, 25.16)	99.6 (98.8, 99.9)	17.8 (17.6, 18.1)	4.63 (1.75, 12.24)	99.0 (97.6, 99.7)	17.8 (17.5, 18.1)
	≥33.3	16	34.99 (11.13, 110.05)	96.6 (95.0, 97.8)	34.6 (34.3, 34.9)	12.66 (5.11, 31.36)	94.4 (91.9, 96.3)	34.7 (34.4, 35.1)
	≥50	25	57.58 (18.33, 180.88)	80.0 (76.9,82.9)	55.0 (54.7, 55.3)	25.73 (10.47, 63.21)	79.5 (75.6, 83.1)	55.0 (54.6, 55.4)
	≥66.6	28	70.62 (22.56, 221.08)	61.7 (58.0, 65.3)	68.9 (68.6, 69.1)	29.15 (11.92, 71.28)	59.3 (54.7, 63.7)	68.7 (64.4, 69.1)
	≥83.3	35	101.72 (32.59, 317.45)	36.9 (33.3, 40.6)	84.2 (83.9, 84.4)	37.31 (15.32, 90.86)	33.6 (29.4, 38.0)	84.2 (83.9, 84.4)
								
Whitehall II Validation: −9, 52	≥16.6	−2	Not applicable			3.82 (0.40, 36.73)	97.4 (86.2, 99.9)	19.1 (17.9, 20.4)
	≥33.3	1				3.73 (0.39, 35.91)	89.5 (75.2, 97.1)	34.0 (32.5, 35.5)
	≥50	8				5.59 (0.65, 47.96)	81.6 (65.7, 92.3)	51.1 (49.5, 52.7)
	≥66.6	20				18.76 (2.48, 142.13)	68.4 (51.3, 82.5)	67.5 (66.0, 68.9)
	≥83.3	27				12.28 (1.56, 95.58)	28.9 (15.4, 45.9)	83.5 (82.3, 84.6)

^a^Reference group lowest category.

## Discussion

In this study, we developed a novel midlife risk score for dementia, CogDrisk-ML, which incorporates contemporary midlife risk factors and can be implemented using the alternative weights for midlife respondents using the existing CogDrisk tool [10] and adding hearing loss as an additional variable. The current version of the CogDrisk assessment tool collects all the information needed to calculate CogDrisk-ML, although the hearing loss item is currently not scored in CogDrisk [18]. Therefore, updating the tool with revised midlife weights makes this an affordable, accessible tool for both individuals and healthcare providers. The integration of CogDrisk-ML with CogDrisk produced the world’s first comprehensive practical dementia risk assessment tool for dementia risk prevention through the life course (midlife to late life) and importantly includes sex-specific weights. The tool also met consumer and clinician demand, with more than two-thirds of the CogDrisk tool users aged 40–64 [20].

Our results show that CogDrisk-ML achieves reasonable *C* statistics across multiple validation cohorts. Similar *C* statistics have been reported for other dementia risk scores, including CogDrisk [11, 15], ANU-ADRI [30], DemNCD risk score [7, 31] and Dementia risk for Chinese type II diabetes [32] when validated using late-life data. In addition, comparison of CogDrisk-ML with existing dementia risk assessment tools such as CAIDE and CogDrisk reveals the better performance of CogDrisk-ML risk scores for middle-aged individuals in order to ascertain their late-life dementia risk.

We, however, observed slightly lower *C* statistics in our study compared to some other UK Biobank-based analyses that developed or validated dementia risk scores [12, 33] using the UK Biobank dataset alone. Several factors could explain these differences. First, our risk factor weights were calculated using a meta-analysis that combined regression coefficients from both the UK Biobank and ARIC studies, and are therefore not specific to any single dataset. Second, both of the previous studies based on UK Biobank cohorts included late-life participants (aged ≥ 65 years), who constitute approximately one-fifth (19.1%) of the UK Biobank sample. In this group, dementia incidence (4.7%) is substantially higher compared to midlife participants (0.7%). Moreover, differences in *C* statistics could stem from variations in sample characteristics between previous analyses and ours. Additional analyses comparing the UK Biobank Dementia Risk Score (UKBDRS) and CogDrisk-ML demonstrated that the performance of the two models across all datasets was similar (see Appendix S5 in the Supplementary Data section). It is important to note that, due to the short follow-up periods, the prevalence of dementia is lower in both the UK Biobank and Whitehall studies compared to the ARIC cohort. Notably, in our analysis, age- and sex-only models performed differently, showing a weaker age–sex effect compared to UKBDRS [13] (see Appendix S5 in the Supplementary Data section for the full details). Further analysis of the UK Biobank data also revealed that modelling age as a continuous vs a categorical variable significantly influenced the Harrell’s *C* statistics (data not shown). Therefore, a proper rigorous comparison of UKBDRS and CogDRisk-ML requires further investigation. Another key consideration is that our primary objective was to develop a dementia risk assessment tool for midlife individuals to predict late-life dementia. To this end, we excluded early-onset dementia cases from our analysis, whereas UKBDRS did not, which may have reduced the predictive ability of the models. This is particularly relevant because the UK Biobank cohort has a relatively low prevalence of dementia (0.7%), which is significantly lower than what would be expected in datasets with longer follow-up periods, where dementia prevalence is typically higher.

In this study, we also conducted a critical analysis of the role of age in dementia risk scoring, particularly when the risk scores were derived from midlife cohorts with short follow-up periods compared with midlife cohorts with long follow-up durations. Our findings show that, in the absence of age and sex effects, CogDrisk-ML, CogDrisk and CAIDE all produced similar *C* statistics across the cohorts. However, for age-only models, cohorts with shorter follow-up periods had similar AUCs to those of full models that included risk and protective factors. In contrast, for long-follow-up midlife cohorts, the age-only models produced notably lower *C* statistics compared to the full models. This might be because of the low prevalence of dementia in the Whitehall and UK Biobank studies due to the fact that only a small number of individuals reached the median age of dementia (~80 years [34]) in these cohorts. These findings warrant further verification with additional long-follow-up datasets from midlife cohorts.

One limitation of our study is that the number of dementia cases in our analysis of the Whitehall dataset is lower than those reported elsewhere [35]. As we obtained the Whitehall II study dataset from the MRC dementias platform UK (DPUK) and are based outside the UK, we are unable to link the dataset with other administrative datasets such as the UK National Hospital Episode Statistics database, the Mental Health Services Data Set, or the mortality register to enhance dementia case astertainment. Due to this limitation, we used the Whitehall II study only for validation of the CogDrisk-ML. We have presented sensitivity and specificity based on quantile cutoffs to help understand the trade-off between sensitivity and specificity for a given cutoff. As the quantile cutoffs may not be optimal, none of the cutoffs produce high sensitivity and specificity. Other studies using a single dataset may have identified the optimal cutoff to maximise sensitivity and specificity. In addition, all three cohorts used in the development of CogDrisk-ML may introduce healthy volunteer bias, which often occurs with cohort studies. Future studies should explore the generalizability of CogDrisk-ML among diverse populations.

## Conclusion

CogDrisk-ML offers a novel, practical approach to dementia risk assessment that will enable the combined CogDrisk to span the entire life course, from midlife to late life, significantly improving prediction accuracy and informing targeted interventions that promote lifestyle changes and reduce dementia risk, ultimately contributing to better health outcomes for individuals at risk.

## Supplementary Material

Appendix_S1_afaf201

Appendix_S2_afaf201

Appendix_S3_afaf201

Appendix_S4_afaf201

Appendix_S5_afaf201

## Data Availability

We have used secondary data analysis. Data may be available from data custodians.
