# Supplementary material for: Development of a midlife-specific CogDrisk algorithm (CogDrisk-ML) to enable validated implementation of dementia risk assessment from midlife to late life
Source: Age Ageing. 2025 Jul 21;54(7):afaf201. doi: 10.1093/ageing/afaf201 (PMC12277239; doi:10.1093/ageing/afaf201)
Supplement: Appendix_S1_afaf201 [file appendix_s1_afaf201.docx]

Appendix S1: Availability and definition of covariates and outcome in various cohorts

| **Covariates** | **Definition** |
| --- | --- |
| Age of the participants | Age at baseline/recruitment. The age range at baseline for the UK Biobank is 50-64 years (UKB field^1^ 21022), while for the ARIC (V1Age01) and Whitehall II cohorts (Age at date of participation wave 5), the age range at baseline is 45-64 years. |
| Sex | Sex as defined in each cohorts. UK Biobank field 31 was used to define sex. |
| Education | For the UK Biobank, education (UKB field 6138) was categorized as follows: a college or university degree, A-levels or equivalent, and NVQ, HND, or HNC or equivalent were classified as "tertiary"; O-levels or equivalent and CSE or equivalent were coded as "secondary"; and "primary" included any other qualification or no qualification.  For the ARIC dataset, variable ELEVEL01, education level definition 1 was used in which education was categorized as: college, graduate school, or professional school as "tertiary"; high school graduate and vocational school as "upper secondary"; and grade school, 0 years of education, or high school without a degree as "less than secondary."  For the Whitehall II study, total year of education: variable ‘tedtotyr’ at wave 5, was categorized as: "less than secondary" (0-8 years), "upper secondary" (9-11 years), and "tertiary" (12 or more years). |
| Obesity | Obesity was defined based on BMI categorization according to WHO guidelines from height and weight in each cohort: underweight (BMI <18.5), normal (BMI 18.5-24.9), overweight (BMI 25-29.9) and obese (BMI >30). The UK biobank field # ‘21001’, ARIC variable: ‘BMI01’ and Whitehall II variable ‘tbmi’ was used for BMI variable. |
| Smoking | Self-reported smoking status were obtained. At the UK Biobank field ‘20116’, ARIC variable ‘CIG01’ cigarette smoking status (variable: ‘tsmkpast’) and Whitehall variable ever smoked cigarettes and currently smoke cigarettes (variable: ‘tsmoke’) was used to defined smoking status. |
| Hypertension | Hypertension was defined based on self-reported mid-life high blood pressure that included both treated and untreated. In the UK Biobank: field ‘2966’: age high blood pressure was detected, for ARIC: Hypertension definition 5 was used (see ARIC codebook for definition) and for the Whitehall II study: self-report of high blood pressure ever told (variable ‘thbp’) and High BP-treatment ever (variable ‘tbpuptrt’). was used to define hypertension, |
| High Cholesterol | Calculated as >6.5 mmol/L or >240 mg/dl. Cholesterol level was obtain from field ‘30690’ from the UK Biobank, Total cholesterol in SI unit from the ARIC: variable ‘TCHSIU01’ and clinical measure of blood cholesterol level at wave 3 (variable: ‘xblchol’) and wave 5 (‘tblchol’). |
| Depression | Defined as frequency of self-reported depressive mode in the UK Biobank (field ‘2966’). For Whitehall II and ARIC dataset CESD scale was used to define depression. We used CESD>8 for ARIC and CESD>20 for the Whitehall II study. |
| Fish serve | Calculated as ≥2 serves per week, where number of fish server were obtain from the UKB field ‘1329’ and dietary intake (DITA34, DITA35, DITA36) at visit 3 in the ARIC study. Average of shellfish (‘tshefish’), whitefish (‘twhifish’) and oily fish (‘toilfish’ per week was obtained from the Whitehall II data. |
| Physical inactivity | Physical activity was scored as the number of self-reported hours of performing activities at each of three intensity levels: mild, moderate and vigorous activities. We used UK Biobank IPAQ activity group (UKB Field ‘22032’) and physical activity level according to Sabia 2011 (‘tmodeme’ & ‘tvigme’) in the Whitehall II study. For ARIC we calculated intensity using MET score based on ARIC variable ‘PAC2’, ‘PAC6’, ‘PAC10’, ‘PAC14’, and then categorize them using standard criteria of intensity and active weekly hours. |
| Cognitive activity | Not available in UK Biobank and ARIC datasets. In the Whitehall study, a form of cognitive activity was available at wave 12 whether individual use internet based activities on TV/radio, music, ebooks, emails. Games and news. Therefore, we didn’t include cognitive activity in our analysis. |
| Diabetes | For ARIC diabetes variable was calculated based on blood glucose>140mg/dl. For UK Biobank (UKB field ‘2443’) and Whitehall II study physician diagnosis of diabetes was used (variable: ever told had diabetes ‘tdiabet’) and ICD 10 codes based definition for long standing diabetes (qlsi_dib). |
| Stroke | Self-reported history of stroke diagnosis was used in the UK Biobank data (UKB field ‘4056’). In the ARIC study, a diagnostic computer algorithm was used to define stroke (‘PRVSTR21’). In the Whitehall clinical diagnosis of stroke was used (‘BT456’). |
| Traumatic brain injury | Defined as self-reported history of brain injury / head injury/ knock out with or without consciousness in ARIC (‘AMHA5’). Head injury status was extracted from non-cancer code ‘1266’ from UKB field ‘20002’). Not available in Whitehall study. |
| Loneliness | Self-reported responses was used in UK Biobank (UKB field ‘2020’). Not available in ARIC. In the Whitehall study, loneliness was defined based on leisure course education (‘tspaedn’, tspaendnf’), leisure culture visit and social visits (‘tspaubf’). |
| Sleep problem | Defined based on self-reported responses in the UK Biobank (UK field ‘1200’). In the Whitehall II study sleep was defined based on Jenkins sleep evaluation questionnaire. Not available in ARIC |
| Hearing loss | Defined based on self-reported hearing impairment in the ARIC (‘Hearing_IMP_RDS’) and self-reported hearing aid use in the Whitehall II study (‘thimpa’). In the UK Biobank, hearing loss was defined based on hearing health outcome and self-related medical conditions (UKB field 2247). |
| Dementia | Algorithms were used for diagnosis of dementia in the ARIC dataset (‘DEMDX3CENS_71’). Self-reported ICD10 codes for longstanding dementia reported any wave after wave 5 in the Whitehall data. For the UK Biobank, we defined dementia based on source of all cause dementia report (UKB field ‘42019’) and date of all cause dementia (‘UKB field ‘42018’). |

Note^1:^ UKB= UK Biobank, <https://biobank.ndph.ox.ac.uk/showcase/field.cgi?id=21022>
