# Supplementary material for: Development of a midlife-specific CogDrisk algorithm (CogDrisk-ML) to enable validated implementation of dementia risk assessment from midlife to late life
Source: Age Ageing. 2025 Jul 21;54(7):afaf201. doi: 10.1093/ageing/afaf201 (PMC12277239; doi:10.1093/ageing/afaf201)
Supplement: Appendix_S2_afaf201 [file appendix_s2_afaf201.docx]

Appendix S2: Descriptive statistics of development and validation samples.

|  | Development | | Validation | | |
| --- | --- | --- | --- | --- | --- |
| Covariates | UK Biobank Study  n=142,010 (%) | ARIC Study  n= 3512 (%) | UK Biobank Study  n= 94,673 (%) | ARIC Study  n= 2338 (%) | Whitehall Study  n=6516 (%) |
| **Age, years (mean, sd)** | 57.5 (4.3) | 52.1 (5.1) | 57.5 (4.3) | 51.7 (5.0) | 55.0 (5.4) |
| **Age group** |  |  |  |  |  |
| 44-49 | NA | 1332 (37.9) | NA | 886 (37.9) | 1439 (22.1) |
| 50-54 | 40546 (28.6) | 1073 (30.6) | 27030 (28.6) | 714 (30.5) | 2026 (31.1) |
| 55-59 | 45793 (32.2) | 739 (21.0) | 30529 (32.2) | 493 (21.1) | 1522 (23.4) |
| 60-64 | 55671 (39.2) | 368 (10.5) | 37114 (39.2) | 245 (10.5) | 1529 (23.5) |
| **Sex** |  |  |  |  |  |
| Male | 63375 (44.6) | 1481 (42.2) | 42248 (44.6) | 985 (42.1) | 4614 (70.8) |
| Female | 78635 (55.4) | 2031 (57.8) | 52425 (55.4) | 1385 (57.9) | 1902 (29.2) |
| **Education** |  |  |  |  |  |
| 0-<8 years | 11164 (7.9) | 485 (13.8) | 7442 (7.9) | 323 (13.8) | 209 (3.2) |
| 8-11 years | 43502 (30.6) | 1480 (42.1) | 29001 (30.6) | 986 (42.2) | 1056 (16.2) |
| >11 years | 87344 (61.5) | 1547 (44.0) | 58230 (61.5) | 1029 (44.0) | 5251 (80.6) |
| **Obesity** |  |  |  |  |  |
| Underweight | 700 (0.5) | 23 (0.7) | 526 (0.6) | 8 (0.3) | 53 (0.8) |
| Normal weight | 46869 (33) | 1266 (36) | 31440 (33.2) | 840 (35.9) | 2760 (42.4) |
| Overweight | 60029 (42.3) | 1431 (40.7) | 39887 (42.1) | 932 (39.9) | 2631 (40.4) |
| Obese | 33779 (23.8) | 791 (22.5) | 22341 (23.6) | 558 (23.9) | 863 (13.2) |
| Missing | 633 (0.4) | * | 479 (0.5) | 0 (0.0) | 209 (3.2) |
| **Smoking status** |  |  |  |  |  |
| Never smoker | 79207 (55.8) | 1690 (48.1) | 52872 (55.8) | 1153 (49.3) | 3253 (49.9) |
| Former smoker | 49647 (35) | 1182 (33.7) | 33050 (34.9) | 793 (33.9) | 2318 (35.6) |
| Current smoker | 12736 (9) | 639 (18.2) | 8448 (8.9) | 390 (16.7) | 693 (10.6) |
| Missing | 420 (0.3) | * | 303 (0.3) | * | 252 (3.9) |
| **Hypertension** |  |  |  |  |  |
| No | 110227 (77.6) | 2634 (75) | 73620 (77.8) | 1759 (75.2) | 5017 (77.0) |
| Yes | 31783 (22.4) | 859 (24.5) | 21053 (22.2) | 567 (24.3) | 1467 (22.5) |
| Missing | 0 (0.0) | 19 (0.5) | 0 (0.0) | 12 (0.5) | 32 (0.5) |
| **High cholesterol** |  |  |  |  |  |
| No | 98866 (69.6) | 2695 (76.7) | 65982 (69.7) | 1837 (78.6) | 4674 (71.7) |
| Yes | 35033 (24.7) | 770 (21.9) | 23354 (24.7) | 480 (20.5) | 1651 (25.3) |
| Missing | 8111 (5.7) | 47 (1.3) | 5337 (5.6) | 21 (0.9) | 191 (2.9) |
| **Depression** |  |  |  |  |  |
| No | 130636 (92) | 3284 (93.5) | 86960 (91.9) | 2206 (94.4) | 5051 (77.5) |
| Yes | 5797 (4.1) | 116 (3.3) | 3914 (4.1) | 63 (2.7) | 395 (6.1) |
| Missing | 5577 (3.9) | 112 (3.2) | 3799 (4) | 69 (3.0) | 1070 (16.4) |
| **Fish** |  |  |  |  |  |
| <2 serve/wk | 9639 (6.8) | 1765 (50.3) | 6470 (6.8) | 1179 (50.4) | 2711 (41.6) |
| >=2 serves/wk | 132170 (93.1) | 1741 (49.6) | 88039 (93) | 1157 (49.5) | 2143 (32.9) |
| Missing | 201 (0.1) | 6 (0.2) | 164 (0.2) | * | 1662 (25.5) |
| **Physical activity** |  |  |  |  |  |
| Less than sufficient | 94721 (66.7) | 1358 (38.7) | 62996 (66.5) | 917 (39.2) | 1266 (19.4) |
| Sufficient | 22999 (16.2) | 2136 (60.8) | 15480 (16.4) | 1405 (60.1) | 5159 (79.2) |
| Missing | 24290 (17.1) | 18 (0.5) | 16197 (17.1) | 16 (0.7) | 91 (1.4) |
| **Diabetes** |  |  |  |  |  |
| No | 134827 (94.9) | 3330 (94.8) | 89836 (94.9) | 2220 (95) | 6323 (97.0) |
| Yes | 6826 (4.8) | 158 (4.5) | 4610 (4.9) | 107 (4.6) | 177 (2.7) |
| Missing | 357 (0.3) | 24 (0.7) | 227 (0.2) | 11 (0.5) | 16 (0.3) |
| **Stroke** |  |  |  |  |  |
| No | 140339 (98.8) | 3489 (99.3) | 93519 (98.8) | 2331 (99.7) | 6478 (99.4) |
| Yes | 1671 (1.2) | 10 (0.3) | 1154 (1.2) | * | 38 (0.6) |
| Missing | 0 (0.0) | 13 (0.4) | 0 (0.0) | * | 0 (0.0) |
| **Traumatic Brain Injury (TBI)** |  |  |  |  |  |
| No | 141405 (99.6) | 3083 (87.8) | 94272 (99.6) | 2064 (88.3) | NA |
| Yes | 605 (0.4) | 402 (11.4) | 401 (0.4) | 256 (10.9) |  |
| Missing | 0 (0.0) | 27 (0.8) | 0 (0.0) | 18 (0.8) |  |
| **Loneliness** |  |  |  |  |  |
| No | 115687 (81.5) | NA | 77006 (81.3) | NA | 4704 (72.2) |
| Yes | 24105 (17) |  | 16105 (17) |  | 1673 (25.7) |
| Missing | 2218 (1.6) |  | 1562 (1.6) |  | 139 (2.1) |
| **Sleep problem** |  |  |  |  |  |
| No | 101203 (71.3) | NA | 67460 (71.3) | NA | 4821 (74.0) |
| Yes | 40650 (28.6) |  | 27125 (28.7) |  | 1589 (24.4) |
| Missing | 157 (0.1) |  | 88 (0.1) |  | 106 (1.6) |
| **Hearing loss** |  |  |  |  |  |
| No | 101767 (74.6) | 2670 (76.0) | 67364 (74.1) | 1767 (75.6) | 6274 (96.3) |
| Yes | 34646 (25.4) | 842 (24.0) | 23562 (25.91) | 571 (24.4) | 133 2.0) |
| Missing | 0 (0.0) | 0 (0.0) | 0 (0.0) | 0 (0.0) | 109 (1.7) |
| **Dementia** |  |  |  |  |  |
| No | 140957 (99.3) | 2796 (79.6) | 93993 (99.3) | 1885 (80.6) | 6464 (99.2) |
| Yes | 1053 (0.7) | 716 (20.4) | 680 (0.7) | 453 (19.4) | 52 (0.8) |
| **Follow-up time (years): mean (sd)** | 12.7 (1.8) | 29.1 (2.42) | 12.7 (1.7) | 29.2 (2.3) | 16.7 (2.8) |

Note: `* ‘indicates a cell of <5 obs
