# Supplementary material for: Development of a midlife-specific CogDrisk algorithm (CogDrisk-ML) to enable validated implementation of dementia risk assessment from midlife to late life
Source: Age Ageing. 2025 Jul 21;54(7):afaf201. doi: 10.1093/ageing/afaf201 (PMC12277239; doi:10.1093/ageing/afaf201)
Supplement: Appendix_S3_afaf201 [file appendix_s3_afaf201.docx]

Appendix S3: Cohort specific and pooled estimated regression coefficient and 95% confidence Intervals (CIs) for males and females.

|  | Male | | | Female | | |
| --- | --- | --- | --- | --- | --- | --- |
| Covariates | ARIC  $\beta(95\% CI)$ | UK Biobank  $\beta(95\% CI)$ | Pooled  $\beta(95\% CI)$ | UK Biobank $\beta(95\% CI)$ | ARIC  $\beta(95\% CI)$ | Pooled  $\beta(95\% CI)$ |
| **Age group** |  |  |  |  |  |  |
| 44-49 | -0.33 (-0.69, 0.02) | NA | -0.33 (-0.69, 0.02) | NA | -0.81 (-1.11, -0.51) | -0.81 (-1.11, -0.51) |
| 50-54 | Ref | Ref | Ref | Ref | Ref | Ref |
| 55-59 | 0.78 (0.47, 1.09) | 2.53 (1.7, 3.36) | 1.61 (-0.10, 3.32) | 4.39 (2.42, 6.36) | 0.45 (0.18, 0.71) | 2.29 (-1.56, 6.15) |
| 60-64 | 1.40 (1.04, 1.76) | 3.91 (3.11, 4.72) | 2.63 (0.16, 5.09) | 5.66 (3.70, 7.63) | 1.23 (0.95, 1.51) | 3.34 (-1.00, 7.68) |
| **Education** |  |  |  |  |  |  |
| 0-<8 years | 0.75 (0.43, 1.07) | 0.44 (0.19, 0.70) | 0.58 (0.28, 0.88) | 0.19 (-0.11, 0.48) | 0.54 (0.25, 0.83) | 0.36 (0.02, 0.71) |
| 8-11 years | 0.39 (0.12, 0.66) | -0.05 (-0.26, 0.15) | 0.16 (-0.27, 0.59) | 0.17 (-0.02, 0.37) | 0.25 (0.02, 0.47) | 0.20 (0.05, 0.35) |
| >11 years | Ref | Ref | Ref | Ref | Ref | Ref |
| **Obesity** |  |  |  |  |  |  |
| Underweight | 0.15 (-1.82, 2.12) | 0.56 (-0.82, 1.94) | 0.43 (-0.70, 1.55) | 0.49 (-0.40, 1.39) | 0.47 (-0.44, 1.37) | 0.48 (-0.16, 1.12) |
| Normal weight | Ref | Ref | Ref | Ref | Ref | Ref |
| Overweight | 0.07 (-0.2, 0.34) | -0.31 (-0.52, -0.10) | -0.13 (-0.50, 0.24) | 0.01 (-0.20, 0.23) | 0.3 (0.08, 0.52) | 0.16 (-0.13, 0.44) |
| Obese | NA | -0.29 (-0.54 ,-0.05) | -0.29 (-0.54, -0.05) | -0.03 (-0.28, 0.23) | NA | -0.03 (-0.28, 0.23) |
| **Smoking status** |  |  |  |  |  |  |
| Never smoker | Ref | Ref | Ref | Ref | Ref | Ref |
| Former smoker | -0.07 (-0.33, 0.19) | -0.03 (-0.21, 0.16) | -0.04 (-0.19, 0.11) | 0.09 (-0.1, 0.29) | 0.04 (-0.21, 0.28) | 0.07 (-0.08, 0.22) |
| Current smoker | 0.2 (-0.14, 0.55) | 0.12 (-0.17, 0.40) | 0.15 (-0.07, 0.37) | 0.31 (-0.03, 0.65) | 0.42 (0.15, 0.69) | 0.38 (0.17, 0.59) |
| **Hypertension** |  |  |  |  |  |  |
| No | Ref |  | Ref |  | Ref |  |
| Yes | 0.24 (-0.02, 0.49) | 0.16 (-0.03, 0.34) | 0.19 (0.03, 0.34) | 0.06 (-0.16, 0.28) | 0.22 (0.00, 0.44) | 0.14 (-0.02, 0.29) |
| **High cholesterol** |  |  |  |  |  |  |
| No | Ref | Ref | Ref | Ref | Ref | Ref |
| Yes | 0.38 (0.12,0.64) | -0.09 (-0.34,0.16) | 0.14 (-0.32, 0.61) | 0.12 (-0.07,0.32) | 0.24 (0.02,0.46) | 0.18 (0.03, 0.33) |
| **Depression** |  |  |  |  |  |  |
| No | Ref | Ref | Ref | Ref | Ref | Ref |
| Yes | 0.39 (-0.25,1.03) | 0.50 (0.11,0.89) | 0.47 (0.14, 0.80) | 0.24 (-0.21,0.69) | 0.54 (0.17,0.9) | 0.42 (0.13, 0.70) |
| **Fish** |  |  |  |  |  |  |
| <2 serves/week | Ref | Ref | Ref | Ref | Ref | Ref |
| >=2 serves/weekk | -0.15 (-0.39,0.09) | -0.44 (-0.73,-0.14) | -0.28 (-0.56, 0.00) | 0.27 (-0.18,0.72) | 0.04 (-0.15,0.24) | 0.08 (-0.10, 0.26) |
| **Physical activity** |  |  |  |  |  |  |
| Less than sufficient | 0.23 (-0.02,0.49) | 0.09 (-0.13,0.31) | 0.15 (-0.01, 0.32) | -0.01 (-0.3,0.28) | 0.09 (-0.12,0.29) | 0.05 (-0.11, 0.22) |
| Sufficient | Ref | Ref | Ref | Ref | Ref | Ref |
| **Diabetes** |  |  |  |  |  |  |
| No | Ref | Ref | Ref | Ref | Ref | Ref |
| Yes | 0.73 (0.31,1.16) | 0.90 (0.66,1.14) | 0.86 (0.65, 1.07) | 0.79 (0.43,1.14) | 0.55 (0.16,0.95) | 0.68 (0.42, 0.95) |
| **Stroke** |  |  |  |  |  |  |
| No | Ref | Ref | Ref | Ref | Ref | Ref |
| Yes | 1.16 (-0.29,2.61) | 1.10 (0.74,1.45) | 1.10 (0.75, 1.45) | 1.08 (0.55,1.62) | -0.51 (-2.48,1.46) | 0.58 (-0.86, 2.03) |
| **Traumatic Brain Injury (TBI)** |  |  |  |  |  |  |
| No | Ref | Ref | Ref | Ref | Ref | Ref |
| Yes | -0.03 (-0.39,0.33) | 0.9 (0.2,1.6) | 0.39 (-0.52, 1.30) | 0.87 (-0.26,2.01) | 0.07 (-0.27,0.41) | 0.28 (-0.41, 0.97) |
| **Loneliness** | NA |  |  |  |  |  |
| No |  | Ref | Ref | NA | Ref | Ref |
| Yes |  | 0.33 (0.1,0.56) | 0.33 (0.1,0.56) |  | 0.32 (0.1,0.55) | 0.32 (0.1,0.55) |
| **Sleep problem** | NA |  |  |  |  |  |
| No |  | Ref | Ref | NA | Ref | Ref |
| Yes |  | 0.19 (0,0.38) | 0.19 (0,0.38) |  | -0.26 (-0.46,-0.06) | -0.26 (-0.46,-0.06) |
| **Hearing loss** |  |  |  |  |  |  |
| No | Ref |  |  |  | Ref |  |
| Yes | 0.10 (-0.15,0.35) | 0.06 (-0.12,0.24) | 0.08 (-0.07, 0.22) | 0.2 (-0.01,0.41) | 0.29 (0.06,0.51) | 0.24 (0.09, 0.39) |
