# Supplementary material for: Development of a midlife-specific CogDrisk algorithm (CogDrisk-ML) to enable validated implementation of dementia risk assessment from midlife to late life
Source: Age Ageing. 2025 Jul 21;54(7):afaf201. doi: 10.1093/ageing/afaf201 (PMC12277239; doi:10.1093/ageing/afaf201)
Supplement: Appendix_S4_afaf201 [file appendix_s4_afaf201.docx]

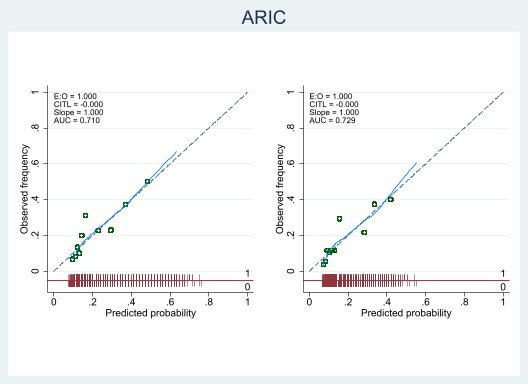


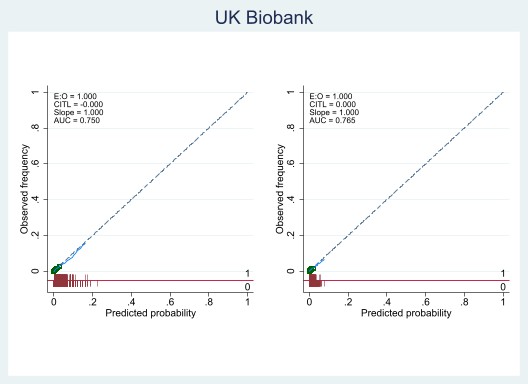


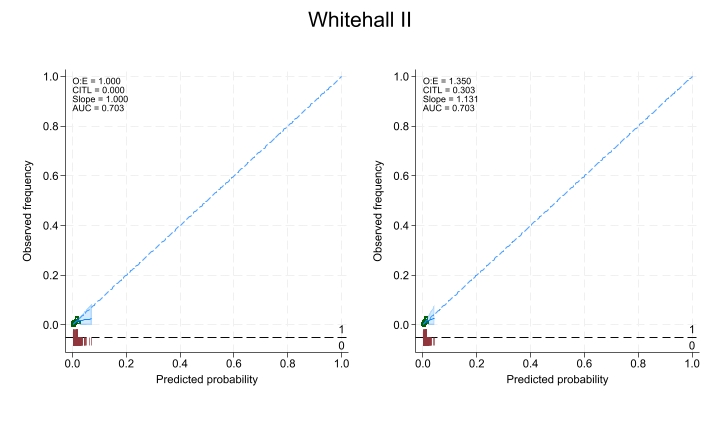


Appendix S4. Calibration plot of observed against expected probabilities for assessment of CogDrisk-ML for predicting dementia.
