# Supplementary material for: Development of a midlife-specific CogDrisk algorithm (CogDrisk-ML) to enable validated implementation of dementia risk assessment from midlife to late life
Source: Age Ageing. 2025 Jul 21;54(7):afaf201. doi: 10.1093/ageing/afaf201 (PMC12277239; doi:10.1093/ageing/afaf201)
Supplement: Appendix_S5_afaf201 [file appendix_s5_afaf201.docx]

*Appendix S5. Comparison Harrell’s C statistics (95% CI) between UKBDRS[1] and CogDrisk-ML across all study cohorts.*

|  | UK-Biobank | | ARIC | | Whitehall II | |
| --- | --- | --- | --- | --- | --- | --- |
| Full Model | CogDrisk-ML | UKBRDS^§^ | CogDrisk-ML | UKBRDS^§^ | CogDrisk-ML | UKBRDS^§^ |
| Male | 0.76 (0.74, 0.79) | 0.77 (0.75, 0.80) | 0.71 (0.68, 0.75) | 0.71 (0.67, 0.75) | 0.71 (0.60, 0.81) | 0.78 (0.68, 0.89) |
| Female | 0.77 (0.74, 0.79) | 0.81 (0.78, 0.83) | 0.73 (0.70, 0.76) | 0.72 (0.69, 0.76) | 0.68 (0.51, 0.85) | 0.70 (0.58, 0.82) |
| Overall | 0.75 (0.73, 0.77) | 0.79 (0.77, 0.81) | 0.71 (0.69, 0.74) | 0.72 (0.69, 0.74) | 0.70 (0.62, 0.79) | 0.75 (0.67, 0.83) |
|  |  |  |  |  |  |  |
| Without age |  |  |  |  |  |  |
| Male | 0.60 (0.56, 0.63) | 0.59 (0.55, 0.63) | 0.63 (0.59, 0.68) | 0.65 (0.61, 0.70) | 0.62 (0.50, 0.73) | 0.52 (0.40, 0.64) |
| Female | 0.57 (0.53, 0.61) | 0.60 (0.56, 0.65) | 0.66 (0.62, 0.70) | 0.65 (0.61, 0.69) | 0.52 (0.34, 0.70) | 0.50 (0.32, 0.69) |
| Overall | 0.59 (0.56, 0.62) | 0.60 (0.57, 0.63) | 0.61 (0.58, 0.64) | 0.64 (0.59, 0.66) | 0.53 (0.42, 0.64) | 0.51 (0.40, 0.62) |
|  |  |  |  |  |  |  |
| Age & sex only model |  |  |  |  |  |  |
| Male | 0.73 (0.71, 0.75) | 0.77 (0.75, 0.79) | 0.66 (0.62, 0.70) | 0.69 (0.65, 0.73) | 0.74 (0.63, 0.84) | 0.79 (0.68, 0.90) |
| Female | 0.75 (0.73, 0.77) | 0.80 (0.77, 0.82) | 0.66 (0.63, 0.70) | 0.70 (0.66, 0.73) | 0.69 (0.56, 0.82) | 0.73 (0.61, 0.84) |
| Overall | 0.72 (0.71, 0.73) | 0.78 (0.77, 0.80) | 0.66 (0.63, 0.69) | 0.69 (0.67, 0.72) | 0.73 (0.65, 0.81) | 0.77 (0.69, 0.85) |

^§^Note: In calculation of UKBDRS, we did not have data on Parental history, Townsend deprivation and Live alone, as we do not have access to these variables in our application of the UK Biobank dataset, and ARIC did not collect information on deprivation. Note that age (years) and education were included in UKBDRS as continuous variable, whereas the CogDrisk-ML included both of them as categorical variable. Clearly, the large difference in between the CogDrisk-ML and UKBDRS are due to how we modelled age.

Reference

[1] Anatürk, M., Patel, R., Ebmeier, K.P., Georgiopoulos, G., Newby, D., Topiwala, A., et al. Development and validation of a dementia risk score in the UK Biobank and Whitehall II cohorts. BMJ Mental Health. 2023;26(1):e300719.
